# Context-specific effects of the identity of detrital mixtures on invertebrate communities

**DOI:** 10.1002/ece3.775

**Published:** 2013-09-17

**Authors:** Melanie J Bishop, Brendan P Kelaher

**Affiliations:** 1 Department of Biological Sciences, Macquarie University 2109New South Wales, Australia; 2 National Marine Science Centre, Southern Cross UniversityPO Box 4321, Coffs Harbour 2450, New South Wales, Australia

**Keywords:** Detritus, environmental context, leaf litter, organic enrichment, spatial subsidy

## Abstract

Many aquatic ecosystems are sustained by detrital subsidies of leaf litter derived from exogenous sources. Although numerous studies have examined the effects of litter species richness and identity on decomposition processes, it remains unclear how these effects extend to associated invertebrate communities or how these effects vary spatially according to local environmental context. Using field enrichment experiments, we assessed how the species richness, assemblage composition, and supply of detrital litter resources interact to affect benthic communities of three temperate Australian estuarine mudflats. Our experiments utilized eight litter sources that are presently experiencing human-mediated changes in their supply to estuarine mudflats. Contrary to predictions, we did not detect effects of the species richness of detrital mixtures on benthic communities. Macroinvertebrate community structure and, in particular, abundance were, instead, influenced by the assemblage composition of detrital mixtures. At two of the three sites, plots receiving the most labile detrital mix, containing the ephemeral algae *Chaetomorpha* and *Ulva*, supported the fewest macroinvertebrates of all the experimental enrichments. The large effect of detrital mix identity on macroinvertebrate communities is of concern given present trends of proliferation of macroalgae at the expense of more refractory seagrasses and marsh grasses. As such environmental degradation continues, it will be important to more fully understand under what environmental contexts such compositional changes in detrital resources will have the most detrimental effects on important prey resources for commercially important fish and wading shorebirds.

## Introduction

The dynamics and food web structure of many ecological systems are determined not by endogenous processes but by the supply of materials, energy, and organisms they receive from other ecosystems (Polis et al. 1997). Exogenously derived resources that alter the dynamics of recipient populations and communities have been termed as spatial subsidies (Polis et al. 1997). Spatial subsidies are highly heterogeneous resources, and their supply to a recipient habitat dependent on the dynamics of the donor system and on transport processes. Consequently, spatial subsidies may arrive at a donor site continuously or in pulses, in large or small volume, and as mixtures or as a single resource (Anderson et al. 2008).

Many aquatic systems are spatially subsidized by leaf litter from other ecosystems (e.g., Fisher and Likens 1972; Richardson 1991; Wallace et al. 1999). Lakes, rivers, and estuaries represent local minima in the vertical relief of the environment. Consequently, these aquatic habitats tend to accumulate organic material that has run off the land, washed down a river from further upstream, or has been transported by waves and currents from other aquatic sites (Polis et al. 1997). Litter is incorporated into surface sediments following shredding and typically decomposes under mixed-species conditions (Anderson and Sedell 1979).

Human activities are increasingly influencing the quality and supply of organic matter inputs to aquatic environments (Macreadie et al. 2012). Range expansions of aquatic and terrestrial producers are adding new litter sources to some localities (e.g., Taylor et al. 2010; Bishop and Kelaher 2013). Local extinctions of donor species are reducing the diversity of litter sources available to others (Bishop et al. 2010). Furthermore, the supply of litter inputs is being modified through alteration of litter transport processes. Construction of dams, storm water drains, seawalls, and groynes can modify the strength of connectivity between terrestrial, freshwater, and coastal ecosystems (e.g., Goodsell 2009; Heatherington and Bishop 2012). Anthropogenic climate change may alter the direction of prevailing winds, the periodicity, and magnitude of rainfall events and the strength of coastal currents that carry litter.

Consequently, how changes in the quality and supply of litter sources impact subsidized food webs is a topic of increasing interest. Many studies have considered how changing the supply and diversity of litter sources impacts decomposition processes in both terrestrial and aquatic environments (reviewed by Gartner and Cardon 2004). Most have shown nonadditive effects of litter mixing on decomposition, but these have differed in direction and magnitude from study to study (Gartner and Cardon 2004; Hättenschwiler et al. 2005), perhaps due to differences in litter quality, methodology, or the decompositional environment (Gartner and Cardon 2004). Few studies have, by contrast, considered how changes in the composition of litter pools may flow on to influence the diversity of associated faunal communities (but see Kelaher and Levinton 2003; Olabarria et al. 2007; Bishop and Kelaher 2008 for examples of those that have). Changes in faunal communities cannot be directly inferred from changes in decomposition rate because some litter constituents contain secondary metabolites, such as tannins, that may negatively affect fauna (Alongi 1987).

Of the studies that have considered the spatial subsidy litter represents to the faunal communities of aquatic habitats, most have considered only the effects of the supply of a single litter source (e.g., Kelaher and Levinton 2003; Olabarria et al. 2007). Several studies have demonstrated effects of different litter species on individual consumers (Duggins and Eckman 1994, 1997), but very few have examined how changes in the composition of detritus affect the structure of whole communities (but see Bishop and Kelaher 2008; Bishop et al. 2010; Olabarria et al. 2010). In addition, it is poorly understood how changes in the supply and quality of litter will vary according to environmental context. Effects of subsidies are likely to vary spatially according to whether they are the sole nutritional source for a community, or supplement a local resource (Polis et al. 1997). Whereas moderate detrital loads may sustain productivity, the supply of large quantities of rapidly decomposing organic material to already enriched environments may induce sediment anoxia and community collapse (Pearson and Rosenberg 1978).

**Figure 1:**
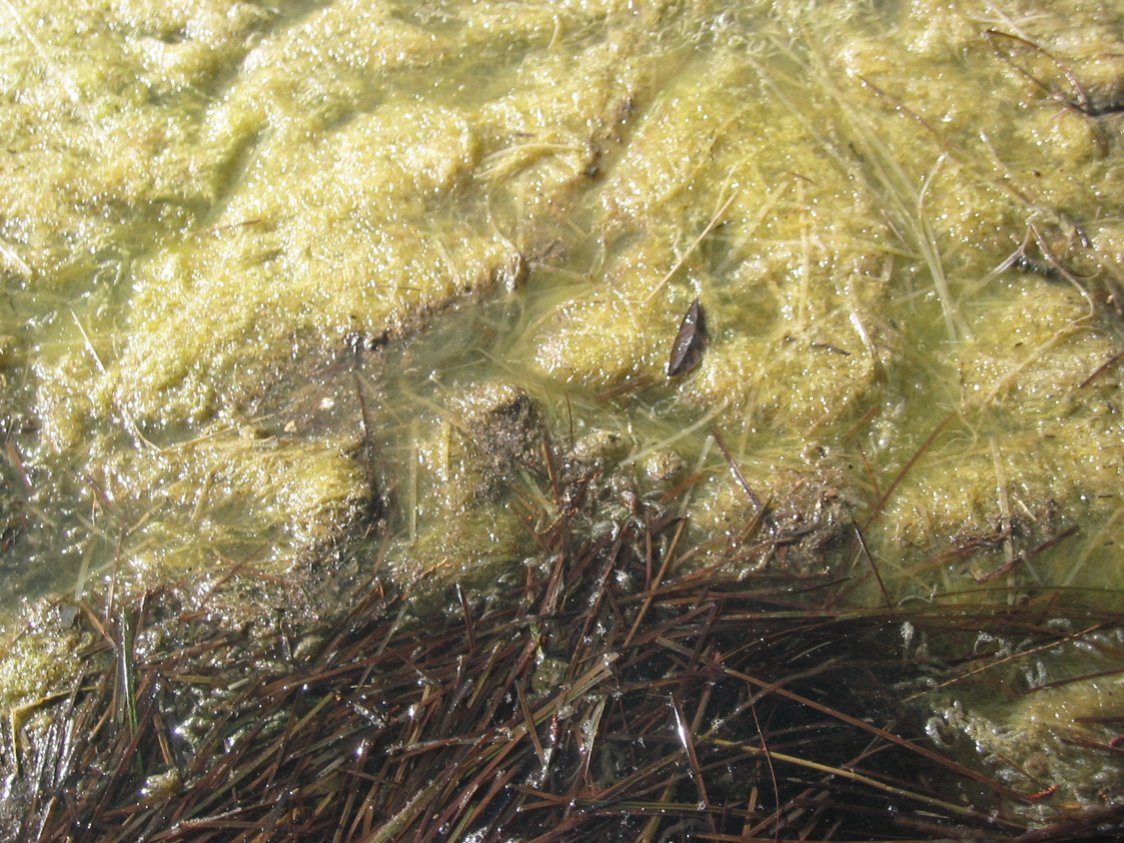
Nutrient enrichment of estuaries can cause overgrowth of seagrasses by fast-growing algae. In Narrabeen Lagoon, New South Wales, Australia, overgrowth of *Zostera muelleri* by *Chaetomorpha* spp. results in an enhancement of the percent contribution of the ephemeral macroalgae to the detrital pool.

Using field enrichment experiments, we assessed how the species richness, identity, and supply of litter resources interact to affect benthic invertebrate communities of temperate Australian estuarine mudflats. Globally, estuaries are currently experiencing significant change in their detrital resources (Fig. 1). Already, over 67% of their wetlands and 65% of their seagrasses have been lost, but overall primary productivity is increasing because of nutrient-stimulated algal blooms (Lotze et al. 2006). We predicted that macroinvertebrates communities, which include functional groups that directly consume detritus and those that consume microalgae stimulated by detrital breakdown (Rublee 1982), would be more abundant and species rich in sediments receiving a greater species richness of phytodetritus because of the greater resource base available. To test the hypothesis that effects of enrichment would be consistent across sites of similar landscape setting, we replicated our experiments across three sites, each situated in a different estuary within the same biogeographical area.

## Materials and Methods

### Study system

The field experiment, manipulating detrital inputs to estuarine sediments, was conducted in Spring 2007 at three locations within a 50 km radius of Sydney, New South Wales (NSW), Australia: Mullet Creek, Hawkesbury River Estuary (33^°^29′33″S, 151^°^15′39″E); Quibray Bay, within Botany Bay (34^°^01′30″S, 151^°^10′45″E); and Grays Point, Port Hacking (34^°^03′59″S, 151^°^05′05″E). The study sites were selected on the basis on their similar landscape setting, which we hypothesized would lead to similar effects of detrital enrichment within each. Each site was within an estuary supporting considerable areas of seagrass and mangrove, with intertidal and shallow subtidal rocky reef, and was adjacent to National Park or Nature Reserve. The selected study sites each comprised of a large, unvegetated, muddy intertidal sandflat and were situated in the mid-lower reaches of estuaries, where the range of the semidiurnal tides is approximately 1.5 m and salinity ranges from 25 to 35 ppt.

Our experiments manipulated the availability of eight major contributors to the detrital biomass of NSW estuaries, each of which is displaying major changes in distribution and abundance. The opportunistic green algae *Ulva* sp. and *Chaetomorpha* sp., and the brown alga *Sargassum* sp. are increasing in abundance as a result of nutrient enrichment, which stimulates their growth, and an increasing area of artificial substrate to which they can attach (M. J. Bishop, pers. obs.). *Caulerpa taxifolia* has recently invaded temperate Australian waters and is now firmly established in at least 14 estuaries and coastal lakes in NSW (Industry and Investment NSW 2009). The gray mangrove, *Avicennia marina,* despite global trends of mangrove loss is transgressing salt marsh in many estuaries (Saintilan and Williams 1999). The seagrasses *Halophila ovalis*, *Zostera muelleri*, and, in particular, *Posidonia australis* are declining due to degradation of habitat and water quality (Shepherd et al. 1989).

At each of the study sites, we established ninety-one 0.25 m^2^ plots for detrital manipulation at a tidal height of MLW springs +0.4 m. The plots, which were separated by a distance of at least 1.5 m, were each marked with a single PVC stake such that they were accessible to benthic predators and other mobile taxa. Each of the plots was randomly assigned to one of 13 treatments (12 detrital manipulations and an undisturbed control treatment).

Our detrital manipulations utilized freshly washed up plant material collected from shores around Sydney. Prior to experimental addition to sediments, it was dried (at 60°C to constant weight) to mimic the natural desiccation of wrack on intertidal shores at low tide and shredded (to <2 mm diameter) because most detritus enters sediments in a particulate form. Addition of detritus to sediments in a dried, shredded form ensured that an equal biomass of detritus was added to replicate plots and that it could be rapidly uptaken by the benthic system. Detritus was added to plots by evenly hand churning it in to the top 0.05 m of sediments at low tide, when the experimental plots are immersed. This method has previously proven effective in manipulating the supply of a variety of detrital resources, with >80% of the enriched material retained by sediments through periods of inundation (e.g., Bishop and Kelaher 2008; Bishop et al. 2010; Taylor et al. 2010). The small spatial scale of detrital manipulation was representative of patchiness on the scale of meters in the accumulation of detritus on intertidal mudflats (Kelaher and Levinton 2003).

### Experimental design

**Figure 2:**
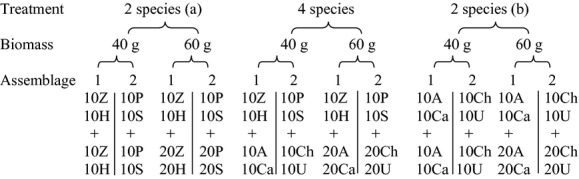
Schematic representation of the experimental design, which separates effects of detrital species richness from effects of biomass and identity of detritus. Letters denote identity of manipulated species (Z = *Zostera muelleri*, H = *Halophila ovalis*, P = *Posidonia australis*, S = *Sargassum* sp., A = *Avicennia marina*, Ca = *Caulerpa taxifolia*, Ch = *Chaetomorpha* sp., U *= Ulva* sp.). Numbers indicate manipulated biomass of species (grams, dry weight).

Our study utilized an experimental design of the type advocated by Benedetti-Cecchi (2004) for unambiguously discriminating among effects of the identity, biomass, and richness of species in biodiversity-ecosystem function experiments (Fig. 2). The design considered two levels of species richness; two and four detrital sources. Although the experimental assemblages were species poor compared with many biodiversity-ecosystem-function experiments, they were representative of the small number of species that typically contribute to the detrital pool at any one location. To ensure that all species of the experimentally manipulated detrital sources occurred in conditions of both high and low species richness, we utilized an additive design that simultaneously controlled for biomass. Our design did not consider detrital monocultures because these rarely occur in nature, are not required by the Benedetti-Cecchi (2004) design, and have formed the basis of previous experiments (Bishop and Kelaher 2008; Bishop et al. 2010).

We randomly assigned four of the eight detrital sources to two assemblages of two species each. To these initial assemblages, in which there was 10 g dry weight of each species, we added either 10 g (low biomass treatment) or 20 g each (high biomass treatment) of two different species (Fig. 2; four species treatment) or, to control for the biomass increase, of the same two species as in the initial assemblage (Fig. 2; 2 species (a) treatment). So as to assess whether any difference in invertebrate communities between the four- and two-species treatments was due to the identity of the additional two species in the higher richness mix, we also established treatments comprising only the two added species (2 species (b)). The outcome was a design with two orthogonal factors, species richness (2 vs. 4) and biomass (40 vs. 60 g), and a third nested factor, assemblage, within species richness. The higher detrital loading was set at 60 g dry weight based on the amount that might reasonably accumulate on estuarine shores following storms (M. J. Bishop, pers. obs.).

In addition to the 12 experimental treatments resulting from our additive design that controlled for biomass (Fig. 2), we also established an undisturbed control treatment. This allowed us to ascertain the impact of detrital additions on benthic assemblages. A disturbance control was unnecessary because the physical disturbance of hand-churning does not detectibly influence either benthic invertebrate abundance (ANOVA: *F*_1,8_ = 2.51, *P* = 0.26) or assemblage composition (PERMANOVA: *F*_1,8_ = 2.62, *P* = 0.25) over a 2-month period. For each of the 13 treatments, we established seven replicate plots.

### Sampling

We assessed effects of experimental treatments on macroinvertebrates in July 2007, two months after detrital addition. A two-month period was appropriate for testing the hypotheses because: (1) it was sufficiently long for changes in benthic communities to occur but any short-term impacts of the physical disturbance of sediments to dissipate (Bishop et al. 2007), (2) it was sufficiently short that any short-term responses of organisms to labile sources would still be evident (Bishop et al. 2010) and that other detrital inputs could be controlled by fortnightly hand removal from plots; and (3) it is the temporal scale on which most variation in macroinvertebrate assemblages occurs in our study system, which is not strongly seasonal (Morrisey et al. 1992).

A single 100-mm-diameter core, of 50-mm depth, was collected from the center of each plot for assessment of macrofaunal communities. The contents of each core were passed over a 500-μm sieve. The animals retained were fixed in 7% formalin for later enumeration to species, or where this was not possible, morphospecies. Using the primary literature (e.g., Beesley et al. 1998, 2000), we assigned each species to a feeding guild: deposit feeder, grazer, shredders/detritivores (hereafter shedders), predators/scavengers (hereafter predators), suspension feeder.

### Statistical analyses

Nonmetric multidimensional scaling (nMDS; PRIMER 6 software, PRIMER-E Ltd., Lutton, Ivybridge, U.K.) of Bray–Curtis dissimilarity measures produced two-dimensional ordinations comparing average assemblage structure among the 12 experimental treatments and 1 control treatment at each site.

Hypotheses about the effects of the richness, assemblage composition, and biomass of detritus on infaunal communities, their total abundance, richness, and abundance of key functional and taxonomic groups were statistically tested using PERMANOVA (Anderson 2001; PRIMER 6 software). The analyses had three factors: treatment (three levels, fixed: four species, two species (a), two species (b)), assemblage (two levels, random: nested in treatment), and biomass (two levels, fixed: 40 g, 60 g). Sites were analyzed separately because in four way analyses, also comparing sites, differences among sites dominated the analysis (PERMANOVA: pseudo-*F*_2,216_ = 993, *P* < 0.001), accounting for over 50% of the variation, and preventing factors of interest from being appropriately tested. Within the factor treatment, preplanned contrasts assessed differences between the two and four species mixes. The control treatment was excluded from PERMANOVA analyses because of the unbalanced experimental design. Analysis of the multivariate community data used Bray–Curtis dissimilarity measures derived from untransformed data. Analyses of the univariate variables, total abundance, richness, and abundance of feeding guilds used Euclidean distances among samples. All analyses used 999 permutations of raw data to assess significance and were followed by *a posteriori* tests to examine sources of significant treatment effects.

The SIMPER (Similarity of Percentages) routine in PRIMER 6 identified species that were important discriminators of macroinvertebrate assemblages among treatments (dissimilarity to standard deviation ratio >1.3, Clarke 1993). Three factor PERMANOVAs, as described above, were also run on these key taxa.

## Results

**Figure 3:**
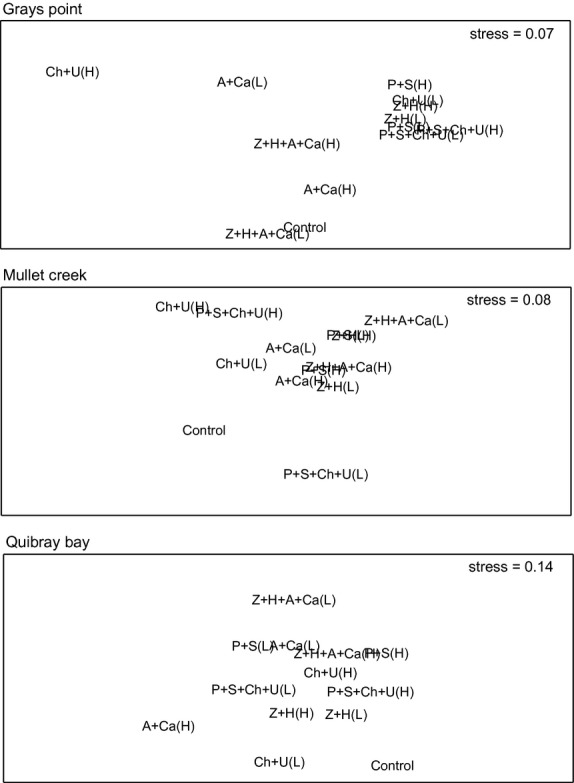
Nonmetric multidimensional scaling ordination of average macroinvertebrate assemblages presents within each of the 13 experimental and control treatments, at each of three locations. *L* = 40 g of detritus added; *H* = 60 g of detritus added. Abbreviations for detrital sources are as given in Fig. 1.

The three study locations differed markedly in the communities of macroinvertebrates they supported (Table 1) and the community-level response of their macroinvertebrates to detrital enrichments (Table 2, Fig. 3). At Grays Point, plots receiving the higher loading of the *Chaetomorpha* and *Ulva* mix supported significantly different macroinvertebrate communities to the other plots which, in turn, did not significantly differ from one another (*a posteriori* tests, sig. Biomass x Assemb with 2 spp. interaction, Table 2; Fig. 3). At Mullet Creek, the assemblage composition of the detrital mixture did not influence macroinvertebrate community structure, but there was a significant effect of the biomass of material added (Table 2; Fig. 3). At Quibray Bay, plots receiving the high biomass of the *Avicennia* and *Caulerpa* mix supported significantly different communities of invertebrates to the other plots, among which communities were statistically indistinguishable (*a posteriori* tests, sig. Biomass × Assemb interaction, Table 2; Fig. 3).

**Table 1:** Summary of the macroinvertebrates collected at each of the three study locations. No. species = total number of species of each group recorded, across all plots. % of abundance = proportionate contribution of each group to total abundance at each site.

	Grays Point		Mullet Creek		Quibray Bay	
			
Bivalves	No. species	% of abundance	No. species	% of abundance	No. species	% of abundance
Bivalves	6	20	5	41	5	19
Gastropods	6	4	7	<1	12	3
Oligochaetes	1	47	1	<1	1	<1
Polychaetes	14	43	8	32	24	60
Amphipods	9	25	7	<1	8	12
Other	8	4	7	26	10	6
Total	44		35		60	

**Table 2:** PERMANOVAs comparing macroinvertebrate assemblages among detrital treatments (Trt; 3 levels, fixed: 4 species, 2 species (a), 2 species (b)), assemblages (Assemblage; 2 levels, random: nested in Treatment), and biomasses (2 levels, fixed: 40 g, 60 g). Terms significant at α = 0.05 are highlighted in bold. *n* = 7.

	df	Grays Point			Mullet Creek			Quibray Bay		
		
		MS	Pseudo-*F*	P	MS	Pseudo-*F*	P	MS	Pseudo-*F*	P
Trt	2	3116	1.82	0.121	1546	1.04	0.459	2388	1.13	0.386
2 vs. 4 spp.	1	1283	0.71	0.831	222	0.11	0.821	2403	1.02	0.660
Among Trts with 2 spp.	1	4949	4.93	0.339	2869	7.65	0.332	2373	0.94	0.667
Assemblage (Trt)	3	1707	1.27	0.170	1492	1.53	0.141	2111	0.92	0.618
Biomass	1	1252	0.65	0.561	3749	4.97	**0.037**	3422	1.01	0.445
Biomass × Trt	2	965	0.50	0.778	916	1.21	0.377	2496	0.74	0.667
Biomass × 2 vs. 4 spp.	1	393	0.21	0.793	1510	2.29	0.208	2698	1.01	0.436
Biomass × Among Trts with 2 spp.	1	1536	0.61	0.623	323	0.44	0.718	2293	0.60	0.807
Biomass × Assemblage (Trt)	3	1925	1.44	0.081	755	0.77	0.665	3382	1.47	**0.036**
Biomass × Assemblage with 2 spp.	2	2536	1.71	**0.050**	740	0.68	0.712	3815	1.64	**0.025**
Residual	72	1339			976			2303		

**Figure 4:**
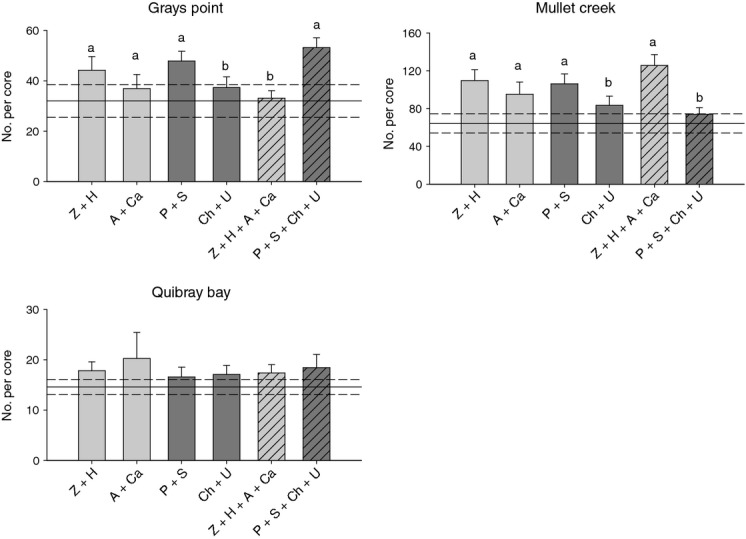
Mean (±1 SE) total abundance of macroinvertebrates in experimental plots receiving two (plain bars) or four (striped bars) species of detritus. Bar colors denote the two-species detrital mixtures that contributed to the same four-species mix. Abbreviations for detrital sources are as given in Fig. 1. Horizontal lines denote the mean (solid line) ±1 SE (broken lines) abundance of macroinvertebrates in physically disturbed, but unenriched, control plots. *n* = 7. Letters denote significant differences among detrital assemblages (*a posteriori* tests, PERMANOVA, Table 3a).

Macroinvertebrate abundance was not influenced by the species richness of detrital mixtures. Instead, at several sites, an effect of the specific assemblage composition of detrital mixtures was seen (sig. Assemb (Trt) effect, Table 3a). At both Grays Point and Mullet Creek, plots receiving the *Chaetomorpha* and *Ulva* mix contained fewer invertebrates than any of the other two-species mixes (*a posteriori* tests: *P* < 0.05, Fig. 4). At Grays Point, a similarly low abundance of macroinvertebrates was also seen in the plots receiving the four-species mix of *Zostera*, *Halophila*, *Avicennia,* and *Caulerpa* (*a posteriori* tests: *P* < 0.05, Fig. 4), and at Mullet Creek, the four-species mix of *Posidonia, Sargassum*, *Chaetomorpha,* and *Ulva* (*a posteriori* tests: *P* < 0.05, Fig. 4). At the third site, Quibray Bay, there was no significant effect of the assemblage composition of detrital mixtures (Table 3a; Fig. 4). The species richness of macroinvertebrates was unaffected by the richness or assemblage composition of detrital mixtures at two of the three locations (Table 3b). At Grays Point, however, we detected a greater species richness of invertebrates in plots receiving the four-species mix of *Posidonia, Sargassum*, *Chaetomorpha,* and *Ulva* than the other treatments (*a posteriori* tests, sig. Assembl (Trt) effect; Table 3b).

**Table 3:** PERMANOVAs comparing the (a) total abundance and (b) species richness of macroinvertebrates among detrital treatments (Trt; 3 levels, fixed: 4 species, 2 species (a), 2 species (b)), assemblages (Assemb; 2 levels, random: nested in Treatment), and biomasses (2 levels, fixed: 40 g, 60 g). *n* = 7. Terms significant at α = 0.05 are highlighted in bold.

	df	Grays Point			Mullet Creek			Quibray Bay		
		
		MS	*F*	P	MS	*F*	P	MS	*F*	P
*(a) Macroinvertebrate abundance*										
Trt	2	57.2	0.59	0.595	24.5	0.37	0.745	15.4	0.52	0.724
2 vs. 4 spp.	1	4.6	0.04	0.811	0.3	<0.01	0.844	<0.1	<0.01	1.000
Among Trts with 2 spp.	1	109.8	24.25	0.327	48.7	9.33	0.323	30.8	0.76	0.657
Assemb (Trt)	3	96.4	3.66	**0.016**	65.7	4.08	**0.009**	29.7	0.28	0.877
Biomass	1	5.2	0.09	0.774	9.9	0.52	0.552	6.0	0.04	0.865
Biomass × Trt	2	34.5	0.62	0.617	2.6	0.14	0.885	163.3	1.06	0.445
Biomass × 2 vs. 4 spp.	1	27.8	0.66	0.502	1.2	0.16	0.756	196.1	2.88	0.245
Biomass × Among Trts with 2 spp.	1	41.3	0.50	0.538	4.0	0.17	0.749	130.5	0.68	0.563
Biomass × Assemb (Trt)	3	55.8	2.12	0.106	19.0	1.18	0.325	154.6	1.45	0.219
Residual	72	26.3			16.1			106.9		
*(b) Macroinvertebrate species richness*										
Trt	2	11.8	0.56	0.729	10.9	4.9	0.134	1.2	0.57	0.609
2 vs. 4 spp.	1	11.5	0.56	0.842	16.1	23.9	0.182	1.0	0.41	0.635
Among Trts with 2 spp.	1	12.1	1.24	0.666	5.8	2.0	0.649	1.4	0.56	1.000
Assemb (Trt)	3	21.1	4.93	**0.006**	2.3	1.1	0.382	2.2	0.26	0.854
Biomass	1	2.0	0.29	0.617	1.0	1.2	0.360	0.1	<0.01	0.928
Biomass × Trt	2	0.2	0.02	0.971	2.3	2.9	0.201	15.8	1.17	0.415
Biomass × 2 vs. 4 spp.	1	<0.1	<0.01	0.931	1.9	2.5	0.270	12.1	0.86	0.478
Biomass × Among Trts with 2 spp.	1	0.3	0.04	0.846	2.6	2.3	0.240	19.4	1.49	0.328
Biomass × Assemb (Trt)	3	6.9	1.62	0.190	0.8	0.4	0.774	13.5	1.63	0.198
Residual	72	4.3			2.1			8.3		

**Figure 5:**
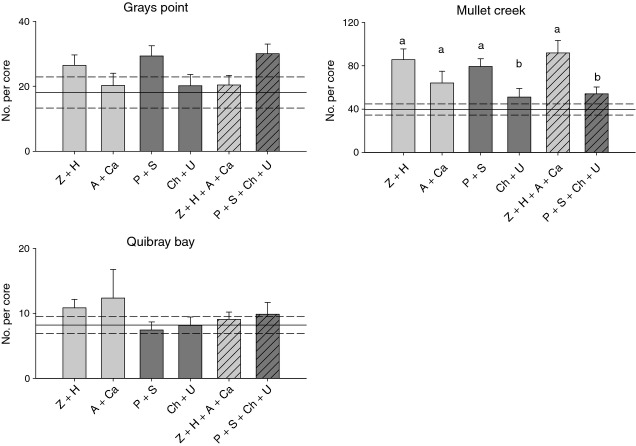
Mean (±1 SE) total abundance of deposit feeders in experimental plots receiving two (plain bars) or four (striped bars) species of detritus. Bar colors denote the two-species detrital mixtures that contributed to the same four-species mix. Abbreviations for detrital sources are as given in Fig. 1. Horizontal lines denote the mean (solid line) ±1 SE (broken lines) abundance of macroinvertebrates in physically disturbed, but unenriched, control plots. *n* = 7. Letters denote significant differences among detrital assemblages (*a posteriori* tests, PERMANOVA, Table 4a).

Analyses revealed few effects of detrital richness, assemblage composition, or biomass on the abundance of macroinvertebrate feeding guilds (Table 4). Of the five guilds examined, only two – the deposit feeders and suspension feeders – displayed a response to the detrital manipulations, and only at specific sites (Table 4a,e). Within Mullet Creek, fewer deposit feeders were found in the plots receiving the two-species mix of *Chaetomorpha* and *Ulva* or the four-species mix of *Posidonia, Sargassum*, *Chaetomorpha,* and *Ulva* than in plots receiving other detrital mixtures (*a posteriori* tests, sig. Assembl effect, Table 4a; Fig. 5). At Grays Point and in Quibray Bay, however, no effect of detrital assemblage composition on deposit feeders was seen (Table 4a; Fig. 5). Suspension feeders displayed a biomass-dependent response to detrital assemblage composition at Quibray Bay, but not at the other two sites (Biomass × Assembl interaction; Table 4e). The source of this interaction could not, however, be resolved with *a posteriori* tests (*P* > 0.05).

**Table 4:** PERMANOVAs comparing the abundance of (a) deposit feeders, (b) grazers, (c) shredders, (d) predators, and (e) suspension feeders among detrital treatments (Trt; 3 levels, fixed: 4 species, 2 species (a), 2 species (b)), assemblages (Assemb; 2 levels, random: nested in Treatment), and biomasses (2 levels, fixed: 40 g, 60 g). *n* = 7. Terms significant at α = 0.05 are highlighted in bold.

	df	Grays Point			Mullet Creek			Quibray Bay		
		
		MS	*F*	P	MS	*F*	P	MS	*F*	P
*(a) Deposit feeders*										
Trt	2	42.5	1.80	0.471	43.9	1.14	0.554	9.1	0.13	0.857
2 vs. 4 spp.	1	2.5	0.08	0.827	1.6	0.03	0.840	1.0	0.10	1.000
Among Trts with 2 spp.	1	82.5	28.87	0.327	86.3	11.72	0.346	17.2	0.17	0.682
Assemb (Trt)	3	23.6	1.68	0.184	38.5	3.37	**0.021**	70.3	1.09	0.386
Biomass	1	5.5	0.16	0.738	37.1	3.42	0.149	15.4	0.19	0.737
Biomass × Trt	2	11.3	0.32	0.735	8.0	0.74	0.543	160.8	2.01	0.276
Biomass × 2 vs. 4 spp.	1	12.5	0.34	0.602	7.6	1.75	0.316	226.3	6.78	0.138
Biomass × Among Trts with 2 spp.	1	10.0	0.20	0.627	8.4	0.61	0.532	95.2	0.82	0.527
Biomass × Assemb (Trt)	3	35.4	2.52	0.072	10.8	0.95	0.413	80.1	1.24	0.297
Residual	72	14.0			11.4			64.4		
*(b) Grazers*										
Trt	2	3.25	3.74	0.287	0.43	12.00	0.210	0.15	1.00	0.540
2 vs. 4 spp.	1	5.36	4.05	0.352	0.86	17.46	0.348	0.29	1.87	0.330
Among Trts with 2 spp.	1	1.14	0.89	0.664	<0.01	<0.01	1.000	0.02	0.11	1.000
Assemb (Trt)	3	0.87	0.59	0.642	0.04	0.11	0.970	0.15	0.82	0.482
Biomass	1	3.44	3.57	0.164	0.01	0.05	0.879	0.30	2.78	0.205
Biomass × Trt	2	2.30	2.38	0.268	0.05	0.21	0.823	0.37	3.44	0.189
Biomass × 2 vs. 4 spp.	1	0.02	0.02	0.888	0.02	0.07	0.840	0.29	3.16	0.233
Biomass × Among Trts with 2 spp.	1	4.57	8.00	0.105	0.07	0.22	0.647	0.45	2.78	0.188
Biomass × Assemb (Trt)	3	0.96	0.65	0.605	0.23	0.72	0.558	0.11	0.57	0.686
Residual	72	1.48			0.31			0.19		
*(c) Shredders*										
Trt	2	2.08	0.81	0.449	13.11	8.74	0.202	2.18	0.25	0.867
2 vs. 4 spp.	1	0.15	0.04	0.844	4.34	2.51	0.173	4.34	0.44	0.669
Among Trts with 2 spp.	1	4.02	3.08	0.358	21.88	27.22	0.328	0.02	<0.01	1.000
Assemb (Trt)	3	2.58	0.45	0.737	1.50	0.47	0.699	8.79	1.96	0.118
Biomass	1	2.68	0.26	0.670	0.05	0.05	0.859	2.33	1.27	0.334
Biomass × Trt	2	6.89	0.68	0.560	2.45	2.45	0.236	2.58	1.41	0.347
Biomass × 2 vs. 4 spp.	1	4.34	0.33	0.643	6.45	6.45	0.126	2.15	0.79	0.470
Biomass × Among Trts with 2 spp.	1	9.45	1.88	0.315	0.01	0.01	0.931	3.02	2.32	0.234
Biomass × Assemb (Trt)	3	10.20	1.79	0.139	0.32	0.32	0.814	1.83	0.41	0.765
Residual	72	5.71			3.17			4.48		
*(d) Predators*										
Trt	2	0.83	0.28	0.862	2.18	8.71	0.185	1.75	0.45	0.865
2 vs. 4 spp.	1	0.95	0.20	0.815	3.34	10.06	0.175	0.48	0.30	0.839
Among Trts with 2 spp.	1	0.71	–	–	0.02	0.20	1.000	3.02	0.52	0.651
Assemb (Trt)	3	5.83	0.44	0.739	0.25	0.26	0.860	3.92	0.74	0.541
Biomass	1	2.98	2.33	0.245	0.11	0.53	0.549	2.68	0.82	0.439
Biomass × Trt	2	18.69	7.48	0.072	1.54	7.56	0.075	2.04	0.63	0.613
Biomass × 2 vs. 4 spp.	1	11.67	9.42	0.092	0.05	0.15	0.722	2.63	0.56	0.520
Biomass × Among Trts with 2 spp.	1	25.71	7.20	0.123	3.02	13.00	0.070	1.44	4.76	0.147
Biomass × Assemb (Trt)	3	2.50	0.37	0.777	0.20	0.21	0.908	3.25	0.61	0.615
Residual	72	6.79			0.96			5.29		
*(e) Suspension feeders*										
Trt	2	88.1	2.01	0.331	165.2	0.39	0.626	6.89	2.51	0.255
2 vs. 4 spp.	1	47.1	0.90	0.635	5.0	0.01	0.822	1.56	0.38	0.659
Among Trts with 2 spp.	1	129.0	9.54	0.318	325.5	15.81	0.345	12.23	4.12	0.313
Assemb (Trt)	3	43.7	1.50	0.217	416.8	1.68	0.169	2.74	0.59	0.656
Biomass	1	24.1	0.62	0.483	874.3	8.06	0.061	2.98	0.18	0.712
Biomass × Trt	2	70.4	1.82	0.283	151.1	1.39	0.379	2.84	0.17	0.850
Biomass × 2 vs. 4 spp.	1	23.6	1.85	0.301	190.7	2.36	0.260	1.19	0.43	0.550
Biomass × Among Trts with 2 spp.	1	117.2	2.09	0.293	111.5	0.97	0.440	4.48	0.19	0.722
Biomass × Assemb (Trt)	3	38.6	1.33	0.253	108.5	0.44	0.744	16.62	3.58	**0.015**
Residual	72	29.1			248.2			4.64		

– no test, denominator of zero.

**Figure 6:**
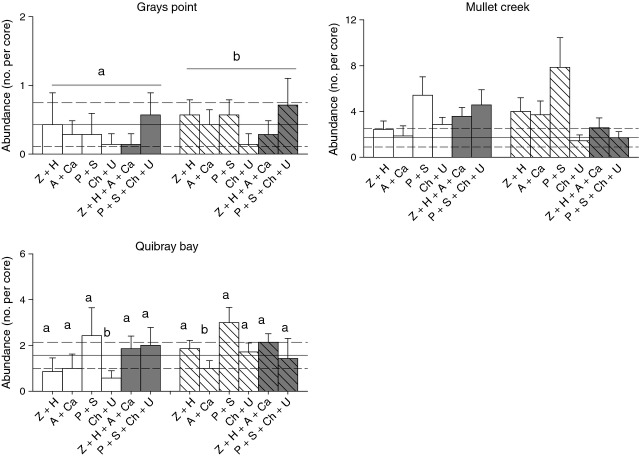
Mean (±1 SE) total abundance of the nereid polychaete *Platynereis* sp. in experimental plots receiving two (white bars) or four (gray bars) species of detritus, to give total detrital biomasses of either 40 g (plain bars) or 60 g (patterned bars) dry weight. Abbreviations for detrital sources are as given in Fig. 1. Horizontal lines denote the mean (solid line) ±1 SE (broken lines) abundance of *Platynereis* sp. in physically disturbed, but unenriched, control plots. *n* = 7. Letters denote significant differences among treatments (*a posteriori* tests, PERMANOVA, Table 5b).

**Figure 7:**
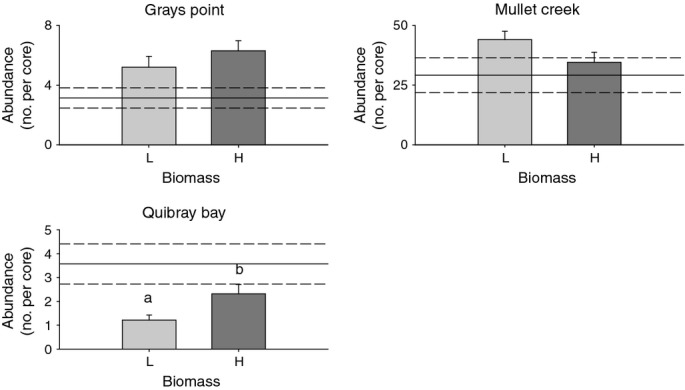
Mean (±1 SE) total abundance of the bivalve *Macomona deltoidalis* in experimental plots receiving a low (L, 40 g; light gray) or high (H, 60 g; dark gray) dry weight of detritus. Horizontal lines denote the mean (solid line) ± 1 SE (broken lines) abundance of *M. deltoidalis* in physically disturbed, but unenriched, control plots. *n* = 7. Letters denote significant differences among biomasses (*a posteriori* tests, PERMANOVA, Table 5c).

SIMPER analysis identified three taxa, the sabellid polychaete *Euchone variabilis,* the nereid polychaete *Platynereis* sp., and the bivalve *Macomona deltoidalis,* as underpinning differences in macroinvertebrate community structure among deterital treatments. At Grays Point and at Mullet Creek, there was no significant effect of the species richness, assemblage composition, or biomass of detritus on *E. variabilis* (Table 5a, Fig. 5). At Quibray Bay, however, the sabellid differed among treatments according to the biomass and mix of detrital material added (sig. Biomass x Assmbl interaction, Table 5a). Differences appeared highly idiosyncratic and could not be differentiated by *a posteriori* tests. At Grays Point, *Platynereis* was more abundant (by 28%) in the highly enriched than the less enriched plots (sig. Biomass effect, Table 5b; Fig. 6), but displayed similar abundance across each of the experimental treatments at Mullet Creek (Table 5b; Fig. 6). At Quibray Bay, there were fewer *Platynereis* in plots receiving the low biomass of *Chaetomorpha* and *Ulva* or the high biomass of *Avicennia* and *Caulerpa* than in the other treatments (*a posteriori* tests, sig. Biomass x Assmbl (Trt) interaction, Table 5b; Fig. 6). *M. deltoidalis* displayed a positive response to increasing detrital enrichment at Quibray Bay (it was 91% more abundant in plots receiving high than low detrital loadings), but not elsewhere (Table 5c, Fig. 7).

**Table 5:** PERMANOVAs comparing the abundance of (a) *Euchone variabilis*, (b) *Platynereis* sp., and (c) *Macomona deltoidalis* among detrital treatments (Trt; 3 levels, fixed: 4 species, 2 species (a), 2 species (b)), assemblages (Assemb; 2 levels, random: nested in Treatment), and biomasses (2 levels, fixed: 40 g, 60 g). *n* = 7. Terms significant at α = 0.05 are highlighted in bold.

		Hacking River			Mullet Creek			Quibray Bay		
				
	df	MS	*F*	P	MS	*F*	P	MS	*F*	P
*(a) Euchone variabilis*										
Trt	2	85.0	2.09	0.355	18.9	0.43	0.595	5.9	1.97	0.314
2 vs. 4 spp.	1	44.0	0.87	0.669	0.3	0.01	0.831	1.5	0.34	0.648
Among Trts with 2 spp.	1	126.0	11.53	0.345	37.5	32.00	0.334	10.3	3.39	0.343
Assemb (Trt)	3	40.7	1.38	0.265	44.2	1.82	0.188	3.0	0.63	0.624
Biomass	1	16.3	0.49	0.533	85.5	8.36	0.064	2.7	0.18	0.734
Biomass × Trt	2	73.0	2.19	0.272	16.0	1.57	0.353	2.7	0.18	0.875
Biomass × 2 vs. 4 spp.	1	20.0	1.49	0.378	20.4	2.83	0.227	0.9	0.32	0.580
Biomass × Among Trts with 2 spp.	1	126.0	2.68	0.250	11.7	1.10	0.404	4.6	0.21	0.720
Biomass × Assemb (Trt)	3	33.3	1.13	0.318	10.2	0.42	0.750	15.3	3.22	**0.020**
Residual	72	29.5			24.3			4.8		
*(b) Platynereis* sp.										
Trt	2	0.37	0.67	0.535	45.8	1.61	0.064	7.37	1.62	0.395
2 vs. 4 spp.	1	0.10	0.12	0.848	6.5	0.49	1.000	1.72	0.43	0.663
Among Trts with 2 spp.	1	0.64	3.60	0.339	85.0	2.00	0.336	13.02	2.00	0.337
Assemb (Trt)	3	0.54	1.28	0.264	28.4	3.23	**0.025**	4.54	1.81	0.159
Biomass	1	0.42	18.00	**0.031**	0.2	0.02	0.904	3.44	2.65	0.221
Biomass × Trt	2	0.04	1.50	0.357	27.1	3.10	0.196	1.65	1.27	0.377
Biomass × 2 vs. 4 spp.	1	<0.01	<0.01	1.000	43.0	7.00	0.117	3.15	2.96	0.238
Biomass × Among Trts with 2 spp.	1	0.07	2.00	0.316	11.2	1.11	0.424	0.16	0.12	0.755
Biomass × Assemb (Trt)	3	0.02	0.06	0.984	8.7	1.00	0.384	1.30	0.52	0.651
Residual	72	0.42			8.8			2.50		
*(c) Macomona deltoidalis*										
Trt	2	2.6	0.29	0.879	14.5	1.1	0.543	0.36	0.10	0.881
2 vs. 4 spp.	1	<0.1	<0.01	1.000	0.5	<0.1	0.814	0.70	0.15	0.455
Among Trts with 2 spp.	1	5.2	0.42	0.666	28.6	10.8	0.316	0.01	<0.01	1.000
Assemb (Trt)	3	8.8	0.39	0.745	12.7	2.1	0.107	3.43	0.75	0.539
Biomass	1	25.2	1.70	0.259	19.1	2.7	0.179	25.37	37.81	**0.009**
Biomass × Trt	2	6.2	0.42	0.670	7.6	1.1	0.432	2.49	3.71	0.148
Biomass × 2 vs. 4 spp.	1	7.3	0.39	0.601	13.3	17.3	0.110	4.95	4.26	0.211
Biomass × Among Trts with 2 spp.	1	5.2	1.03	0.441	1.9	0.2	0.768	0.02	0.02	0.876
Biomass × Assemb (Trt)	3	14.8	0.66	0.578	7.1	1.2	0.301	0.67	0.15	0.937
Residual	72	22.4			6.1			4.53		

## Discussion

Previous studies have demonstrated nonadditive effects of litter mixing on the decomposition of detrital material (Gartner and Cardon 2004; Hättenschwiler et al. 2005). Our study sought to provide one of the first assessments in an estuarine setting of whether nonadditive effects of species mixing extend to the macroinvertebrate communities subsidized by this resource. We predicted that there would be a greater abundance and richness of macroinvertebrates in the plots receiving the 4-species than the 2-species mixtures of macrophytic detritus due to the broader resource base available in more species-rich mixtures. These communities include functional groups that directly consume detritus and that consume microalgae stimulated by detrital breakdown (Rublee 1982). Contrary to predictions effects of detrital species richness on macroinvertebrate assemblage structure, total macroinvertebrate abundance and species richness were not detected at any of the three sites. Instead, macroinvertebrate community structure displayed responses to the identity of detrital assemblages and to detrital loading that varied among sites.

At all three sites, aspects of macroinvertebrate community structure differed between the plots receiving the most labile mix of *Chaetomorpha* sp. and *Ulva* sp. and the plots receiving the other two-species mixtures. Among sites, however, the strength and source of the difference varied. At two of the sites, differences in macroinvertebrate communities among plots receiving *Chaetomorpha* sp. and *Ulva* sp., and the other two-species mixes were underpinned by an overall lower abundance of invertebrates in the plots receiving the labile detrital mix. At one of these sites, the lower overall abundance of invertebrates in the *Chaetomorpha* sp. and *Ulva* sp. treatment was due to fewer deposit feeders, but at the other site, the source of this difference in abundance was unclear. At the third site, only one taxon, the detritivorous polychaete *Platynereis,* responded differentially to the *Chaetomorpha* and *Ulva* mix, being less abundant in this the other treatments. At Mullet Creek, the 4-species mix of *Posidonia*, *Sargassum*, *Chaetomorpha,* and *Ulva* also contained fewer invertebrates than the other treatments.

Labile detritus is much more readily decomposed by microbial assemblages than refractory resources that have a higher C/N ratio and fiber content (Melillo et al. 1982; Hobbie 2005). We suspect that the generally smaller abundances of invertebrates, and in particular deposit feeders, in the *Chaetomorpha* and *Ulva* mix may be because this detritus was very rapidly decomposed and assimilated, such that it did not continue to provide an enhanced carbon and nutrient supply over the two-month duration of our study. Whereas detrital enrichment generally enhanced macroinvertebrate abundance over background levels in unenriched sediments, the plots receiving the *Chaetomorpha* and *Ulva* mix generally had abundances more closely matching the control treatment. Particulate detritus was notably absent from sediments receiving the *Chaetomorpha* and *Ulva* mix at the end of the experiment, but was still present in sediments receiving more refractory resources. Previous research has found that on its own, *Ulva* sp. detritus, which has a half-life of 8-12 days, leaves no lasting impact on macroinvertebrates over a 1-month period, when supplied as a pulse input (Rossi 2006). In the 4-species mixtures, *Chaetomorpha* and *Ulva* may accelerate the decomposition of the more refractory resources. Synergistic effects of litter mixing on decomposition have been hypothesized to result from transfer of decomposition-enhancing nutrients from high- to poor-quality litter components (Gartner and Cardon 2004).

At Grays Point, we also detected a smaller overall abundance of macroinvertebrates in the plots receiving the 4-species mixture of *Zostera*, *Halophila*, *Avicennia,* and *Caulperpa* than in the other treatments. Macroinvertebrate abundance in this treatment was similar to in plots receiving the high loading of *Chaetomorpha* and *Ulva*. The few macroinvertebrates supported by this 4-species mix may be explained by the lability of the *Caulerpa* (C/N 17.1 ± [1 SE] 0.7, *n* = 2; c.f. 22.9 ± 0.1 for *Zostera*, 26.3 ± 0.1 for *Avicennia,* and 20.7 ± 0.1 for *Halophila*)*,* accelerating decomposition of the litter mixture and producing sediment anoxia through microbial activity. Alternatively, the pattern may reflect chemical deterrence of fauna by the secondary metabolites contained within *Avicennia marina* and *Caulepra taxifolia*. *Avicennia* contains tannins that, although rapidly leached from senesced mangrove leaves, can remain in sediments and deter fauna for extended periods by binding to silt and lay particles (Alongi 1987). *C. taxifolia* contains caulerpenyne that deters herbivores (Gollan and Wright 2006) and possibly also detritivores (Taylor et al. 2010; Bishop and Kelaher 2013). At Quibray Bay, the high loading of *Avicennia* and *Caulerpa* significantly modified invertebrate community structure, by reducing the abundance of the detritivore, *Platynereis* sp.

Effects to macroinvertebrate communities of detrital loading were less pervasive than effects of detrital assemblage identity, differing among sites and taxa. At Grays Point, the detritivore *Platynereis* was more abundant in plots receiving the low than the high detrital load, but at Quibray Bay, the deposit-feeding bivalve *M. deltoidalis* displayed the reverse pattern. The species-specific impacts of loading suggest that in this study, its effect was not mediated by overall environmental deterioration at high supply. If high loading had stimulated sediment anoxia through rapid bacterial breakdown of excessive organic matter, negative impacts would be expected among many of the subsurface taxa (see Bishop and Kelaher 2013). Instead, taxa may be displaying individualistic responses to alteration of resource supply, microbial communities, or sediment chemistry.

Although our study sites were carefully selected to be climatically and ecologically similar, they nevertheless differed from one another in several ways that may have influenced detrital impacts. Although all three were situated in sheltered estuaries of the greater Sydney metropolitan area and were chosen for their similar landscape context, they were each situated in different catchments of varying degree of urbanization, their sediment grain size differed (Mullet Creek was the coarsest and Grays Point, the finest), as did their baseline benthic communities (see Table 1). These factors, and others, may have independently or interactively mediated identity effects. Detritivore diversity and identity can influence litter decay processes (Srivastava et al. 2009; Vos et al. 2010) which, in turn, feedback to influence invertebrate communities. Sediment grain size can mediate effects of disturbance on estuarine macrobenthic communities (Lindegarth and Hoskin 2001), and background organic enrichment clearly plays a role (Pearson and Rosenberg 1978). It is clear that a better grasp of underlying mechanisms impacted by detrital species richness and identity are needed to understand the context dependency of the relationship.

Overall, our results add to growing evidence (e.g., Bishop and Kelaher 2008; Olabarria et al. 2010) that the identity of detrital material is a far more important determinant of its effect on macroinvertebrates than species richness. This result parallels the finding that detrital source richness does not have an overt effect on litter decay processes, but instead, there are important idiosyncratic effects that flow on from litter mixing (Smith and Bradford 2003, Moore and Fairweather 2006). Although detrital decomposition rates will undoubtedly influence macroinvertebrate community composition, litter chemistry, independent of effects on decomposition rate, may also play an important role by influencing palatability (e.g., Alongi 1987). Trait-based studies are needed to develop general rules for when and where changes to detrital species pools have positive versus negative effects on invertebrate productivity.

The failure of high loadings of labile detritus to support dense invertebrate communities is of concern due to the important prey base these provide to fish and shorebirds, and the shifting composition of detrital pools. Habitat destruction, global climate change, pollution, and species invasions are increasingly modifying the distribution and abundance of terrestrial and aquatic primary producers (e.g., Ashton et al. 2005; Harley et al. 2006; Waycott et al. 2009), often resulting in shifts in detrital pools from more refractory to labile resources (e.g., Bishop et al. 2010; Bishop and Kelaher 2013). Given that our results indicate that negative impacts of over-enrichment of sediments with labile detritus are common, but not pervasive, the challenge is now to determine under what circumstances they will be most detrimental so that appropriate strategies for managing this environmental change may be put in place.

## References

[b1] Alongi DM (1987). The influence of mangrove derived tannins on intertidal meiobenthos in tropical mangrove estuaries. Oecologia.

[b2] Anderson MJ (2001). A new method for non-parametric multivariate analysis of variance. Austral Ecol.

[b3] Anderson NH, Sedell JR (1979). Detritus processing by macroinvertebrates in stream ecosystems. Annu. Rev. Entomol.

[b4] Anderson WB, Wait DA, Stapp P (2008). Resources from another place and time: responses to pulses in a spatially subsidised system. Ecology.

[b5] Ashton IW, Hyatt LA, Howe KM, Gurevitch J, Lerdau MT (2005). Invasive species accelerate decomposition and litter nitrogen loss in a mixed deciduous forest. Ecol. Appl.

[b6] Beesley PL, Ross GJB, Wells A (1998). Mollusca: the southern synthesis. Fauna of Australia, volume 5, parts A and B.

[b7] Beesley PL, Ross GJB, Glasby CJ (2000). Polychaetes and allies: the southern synthesis. Fauna of Australia, volume 4A. Polychaeta, Myzostomida, Pogonophora, Echiura, Sipuncula.

[b8] Benedetti-Cecchi L (2004). Increasing accuracy of causal inference in experimental analyses of biodiversity. Funct. Ecol.

[b9] Bishop MJ, Kelaher BP (2008). Non-additive, identity-dependent effects of detrital species mixing on soft-sediment communities. Oikos.

[b10] Bishop MJ, Kelaher BP (2013). Replacement of native seagrass with invasive algal detritus: impacts to estuarine sediment communities. Biol. Invasions.

[b11] Bishop MJ, Kelaher BP, Alquezar RA, York PH, Ralph PJ, Skilbeck CG (2007). Trophic cul-de-sac, *Pyrazus ebeninus*, limits trophic transfer through an estuarine detritus-based food web. Oikos.

[b12] Bishop MJ, Coleman MA, Kelaher BP (2010). Cross-habitat impacts of species decline: response of estuarine sediment communities to changing detrital resources. Oecologia.

[b14] Clarke KR (1993). Non-parametric multivariate analyses of changes in community structure. Aust. J. Ecol.

[b15] Duggins DO, Eckman JE (1994). The role of kelp detritus in the growth of benthic suspension feeders in an understory kelp forest. J. Exp. Mar. Biol. Ecol.

[b16] Duggins DO, Eckman JE (1997). Is kelp detritus a good food for suspension feeders? Effects of kelp species, age and secondary metabolites. Mar. Biol.

[b17] Fisher SG, Likens GE (1972). Stream ecosystem: organic energy budget. Bioscience.

[b18] Gartner TB, Cardon ZG (2004). Decomposition dynamics in mixed-species leaf litter. Oikos.

[b19] Gollan JR, Wright JT (2006). Limited grazing pressure by native herbivores on the invasive seaweed *Caulerpa taxifolia* in a temperate Australian estuary. Mar. Freshw. Res.

[b20] Goodsell PJ (2009). Diversity in fragments of artificial and natural marine habitats. Mar. Ecol. Prog. Ser.

[b21] Harley CDG, Hughes AR, Hultgren KM, Miner BG, Sorte CJB, Thornber CS (2006). The impacts of climate change in coastal marine systems. Ecol. Lett.

[b22] Hättenschwiler S, Tiunov AV, Scheu S (2005). Biodiversity and litter decomposition in terrestrial ecosystems. Annu. Rev. Ecol. Evol. Syst.

[b23] Heatherington C, Bishop MJ (2012). Spatial variation in the structure of mangrove forests with respect to seawalls. Mar. Freshw. Res.

[b24] Hobbie SE (2005). Contrasting effects of substrate and fertilizer nitrogen on the early stages of litter decomposition. Ecosystems.

[b25] Industry and Investment NSW (2009). NSW control plan for the noxious marine alga Caulerpa taxifolia.

[b27] Kelaher BP, Levinton JS (2003). Variation in detrital enrichment causes spatio-temporal variation in soft-sediment assemblages. Mar. Ecol. Prog. Ser.

[b28] Lindegarth M, Hoskin M (2001). Patterns of distribution of macro-fauna in different types of estuarine soft sediment habitats adjacent to urban and non-urban areas. Estuar. Coast. Shelf Sci.

[b30] Lotze HK, Lenihan HS, Bourque BJ, Bradbury RH, Cooke RG, Kay MC (2006). Depletion, degradation, and recovery potential of estuaries and coastal seas. Science.

[b32] Macreadie PI, Allen K, Kelaher BP, Ralph PJ, Skilbeck CG (2012). Paleoreconstruction of estuarine sediments reveal human-induced weakening of coastal carbon sinks. Glob. Change Biol.

[b33] Melillo JM, Aber JD, Muratore JF (1982). Nitrogen and lignin control of hardwood leaf litter decomposition dynamics. Ecology.

[b501] Moore TN, Fairweather PG (2006). Decay of multiple species of seagrass detritus is dominated by species identity, with an important influence of mixing litters. Oikos.

[b34] Morrisey DJ, Howitt L, Underwood AJ, Stark JS (1992). Temporal variation in soft sediment benthos. J. Exp. Mar. Biol. Ecol.

[b35] Olabarria C, Lastra M, Garrido J (2007). Succession of macrofauna on macroalgal wrack of an exposed sandy beach: effects of patch size and site. Mar. Environ. Res.

[b36] Olabarria C, Incera M, Garrido J, Rossi F (2010). The effect of wrack composition and diversity on macrofaunal assemblages in intertidal marine sediments. J. Exp. Mar. Biol. Ecol.

[b37] Pearson TH, Rosenberg R (1978). Macrobenthic succession in relation to organic enrichment and pollution of the marine environment. Oceanogr. Mar. Biol. Annu. Rev.

[b38] Polis GA, Anderson WB, Holt RD (1997). Towards an integration of landscape and food web ecology: the dynamics of spatially subsidized food webs. Annu. Rev. Ecol. Syst.

[b39] Richardson JS (1991). Seasonal food limitation of detritivores in a montane stream: an experimental test. Ecology.

[b40] Rossi F (2006). Small-scale burial of macroalgal detritus in marine sediments: effects of Ulva spp. on the spatial distribution of macrofauna assemblages. J. Exp. Mar. Biol. Ecol.

[b41] Rublee PA (1982). Seasonal distribution of bacteria in salt-marsh sediments in North Carolina. Estuar. Coast. Shelf Sci.

[b42] Saintilan N, Williams RJ (1999). Mangrove transgression into saltmarsh environments in south-east Australia. Glob. Ecol. Biogeogr.

[b43] Shepherd SA, McComb AJ, Bulthius DA, Neverauskas V, Steffensen DA, West R, Larkum AWD, McComb AJ, Shepherd SA (1989). Decline of seagrasses. Biology of seagrasses.

[b502] Smith VC, Bradford MA (2003). Do non-additive effects on decomposition in litter-mix experiments result from differences in resource quality between litters?. Oikos.

[b45] Srivastava DS, Cardinale BJ, Downing AL, Duffy JE, Jouseau C, Sankaran M (2009). Diversity has stronger top-down than bottom-up effects on decomposition. Ecology.

[b46] Taylor SL, Bishop MJ, Kelaher BP, Glasby TM (2010). Impacts of detritus of the invasive alga *Caulerpa taxifolia* on a soft sediment community. Mar. Ecol. Prog. Ser.

[b47] Vos VC, Berg AJ, van Ruijven MP, Peeters ETHM, Berendse F (2010). Macro-detritivore identity drives leaf litter diversity effects. Oikos.

[b48] Wallace JB, Eggert SL, Meyer JL, Webster JR (1999). Effects of resource limitation on a detrital-based ecosystem. Ecol. Monogr.

[b49] Waycott M, Duarte CM, Carruthers TJB, Orth RJ, Dennison WC, Olyarnik S (2009). Accelerating loss of seagrasses across the globe threatens coastal ecosystems. Proc. Natl. Acad. Sci. USA.

